# Impact of Impulse Control Disorders on Sleep-Wake Regulation in Parkinson's Disease

**DOI:** 10.1155/2015/970862

**Published:** 2015-11-18

**Authors:** Atbin Djamshidian, Werner Poewe, Birgit Högl

**Affiliations:** Department of Neurology, Medical University of Innsbruck, 6020 Innsbruck, Austria

## Abstract

Sleep disturbances are common in patients with Parkinson's disease (PD) and are even more prevalent in patients with behavioural addictions, such as pathological gambling, compulsive sexual behaviour, compulsive buying, binge eating, punding, and the compulsive use of dopamine replacement therapy. An overview of the relationship between these impulse control disorders and sleep disturbances is given and potential underlying mechanisms and treatment strategies are covered.

## 1. Introduction

Impulsivity has been described as “a behaviour that is performed with little or inadequate forethought” [1] or as a failure to “resist an impulse.” In Parkinson's disease (PD) impulse control disorders (ICDs), such as gambling disorder, compulsive shopping, binge eating, compulsive sexual disorder, and punding and dopamine dysregulation syndrome, are an increasingly well-recognised adverse-effect of dopaminergic medication, in particular dopamine agonists. Initial studies suggested that these problems arise in about 14% of treated patients [2], but more recent studies indicate that up to 40% of PD patients may be affected [3]. This discrepancy may be explained because ICD symptoms are underdiagnosed in clinical practise. Furthermore, much higher prevalence rates of ICDs are found when screening is performed with a caregiver or family member rather than with the PD patient alone [4] likely because patients often do not disclose aberrant behaviours due to shame or lack of insight [5].

It is unclear why only a subgroup of treated PD patients develops ICDs and others do not. This suggests that there are protective factors to prevent people from addiction, possibly of genetic origin [6]. Apart from dopamine replacement therapy other risk factors for developing ICDs in PD include younger onset of disease, higher novelty seeking personality traits, and a personal or family history of addictive behaviours [7]. Furthermore, several studies have suggested a relationship between sleep disturbances and ICDs in PD [8–10]. The purpose of this manuscript is to explore this relationship in more detail. A PubMed literature review searching for the terms “Impulse control disorders, Parkinson's disease, Impulsive compulsive behaviours, sleep disorders in Parkinson's disease, Sleep and Impulsivity” was carried out until May 2015; however, this is a narrative review with potential selection bias.

## 2. Impulse Control Disorders in PD

Behavioural addictions in PD include pathological gambling, compulsive sexual disorder, binge eating, compulsive shopping, the dopaminergic dysregulation syndrome (the overuse of dopaminergic medication), and punding, a stereotype, repetitive, purposeless behaviour driven by fascination. Although PD patients may exhibit only one addictive behaviour, at least a quarter has two or more addictions [2]. A brief summary of the most common behavioural addictions is given below.

### 2.1. Pathological Gambling

Pathological gambling has been now reclassified as gambling disorder and is defined as an inappropriate, persistent, and maladaptive behaviour. Typically PD patients prefer gambles that are repetitive, require little higher cortical processing, and have high reward uncertainty [12] and brief loss periods. These include slot machines, lottery/scratch cards, and internet gambling, such as roulette which offers “near misses,” and losses can be instantly chased. PD patients sometimes develop complex ritualistic behaviours such as lucky charms prior to gambling [13] and may develop loss chasing behavior. Diagnostic criteria of gambling disorder from DSM-V [11] are as follows:

- Persistent and recurrent problematic gambling behavior leading to clinically significant impairment or distress, as indicated by the individual exhibiting four (or more) of the following in a 12-month period:
  1. Needs to gamble with increasing amounts of money in order to achieve the desired excitement.
  2. Is restless or irritable when attempting to cut down or stop gambling.
  3. Is often preoccupied with gambling (e.g., having persistent thoughts of reliving past gambling experiences, handicapping or planning next venture, and thinking of ways to get money with which to gamble).
  4. Often gambles when feeling distress (e.g., helpless, guilty, anxious, and depressed).
  5. Often returns another day to get even (“chasing” one's losses), After losing money gambling.
  6. Lies to conceal the extent of involvement with gambling.
  7. Has jeopardized or lost a significant relationship, job, or educational career opportunity because of gambling.
  8. Relies on others to provide money to relieve desperate financial situations caused by gambling.
- The gambling behavior is not better accounted for by a manic episode.

### 2.2. Compulsive Sexual Behaviour

The prevalence of compulsive sexual behaviour in PD has been found to be 3.5% [2] although these rates vary depending on cultural difference. Furthermore, standardized criteria are missing. It is, however, likely that this ICD is far more common as patients and their partners are often too embarrassed to declare the problem. Typically compulsive sexual behaviour is more problematic in males. Other phenomena such as zoophilia and paraphilia are much rarer but have also been described in PD [14, 15].

### 2.3. Punding

Punding is defined as a stereotype, repetitive, and nongoal orientated behaviour. It was first described in the 1970s in amphetamine and cocaine addicts [16] and much later in PD [17]. Typically, punding is idiosyncratic [18]. For example, men tend to tinker more often with technical equipment whereas women prefer sorting and cleaning. The prevalence rate for punding varies widely from 1.4% to 14%. Whether punding is more common in patients with cognitive decline is currently unknown.

Hobbyism, reckless generosity [19], hoarding [20], drug addiction [21], and compulsive smoking [22] are other albeit less reported phenomena.

### 2.4. Dopamine Dysregulation Syndrome (DDS)

DDS is characterised by a compulsive overuse of typically fast acting dopaminergic medication, such as dispersible levodopa. Patients with DDS often take extra medication in order to avoid the unpleasant “off” periods despite the adverse consequences such as dyskinesia. Punding is frequently seen in this cohort of patients [23]. Estimates for DDS in PD range from 0.6% to 4% [24, 25]. Furthermore, new onset of DDS can occur in PD patients with dopamine agonist withdrawal syndrome in an attempt to improve their symptoms [26]. Diagnostic criteria for DDS [24] are as follows:

- Parkinson's disease with documented L-dopa responsiveness.
- Need for increasing doses of dopamine replacement therapy (DRT) in excess of those normally required to relieve parkinsonian symptoms and signs.
- Pattern of pathological use: expressed need for increased DRT in the presence of excessive and significant dyskinesias despite being “on,” drug hoarding, drug seeking behaviour, unwillingness to reduce DRT, and absence of painful dystonias.
- Impairment in social or occupational functioning: fights, violent behaviour, loss of friends, absence of work, loss of job, legal difficulties, and arguments or difficulties with family.
- Development of hypomanic, manic, or cyclothymic affective syndrome in relation to DRT.
- Development of a withdrawal state characterized by dysphoria, depression, irritability, and anxiety on reducing the level of DRT.
- Duration of disturbance for at least 6 months.

## 3. Mechanisms Underlying ICDs in PD

While the dopamine dysregulation syndrome, punding, and also walkabouts are commonly associated with levodopa, dopamine agonists are more likely to trigger the other addictive behaviours (e.g., compulsive shopping, gambling disorder, compulsive sexual disorder, and binge eating). The exact underlying pathophysiology of ICDs in PD remains elusive. It is, however, possible that differences in dopamine receptor stimulation play a key role in triggering these behaviours. Dopamine agonists mainly stimulate dopamine D2 and D3 receptors which are mainly located in the ventral striatum. Furthermore, dopamine agonists can cause neuroplastic changes in susceptible patients causing increased dopamine release in the ventral striatum to reward related cues [27, 28] and sensitization of the ventral striatum [27]. These elevated mesolimbic dopamine levels are thought to cause impaired decision making and risky choices and may play a major role in driving addictive behaviours [29]. Addiction can become habitual and compulsive [30, 31] which means that behaviourally the outcome of an action is becoming less important. Instead, the patients' decision making shifts from a “goal directed” to a “stimulus-response” behaviour in which the stimulus (and not an outcome) drives an action [32]. Thus, habit formation requires little cortical activity as the behaviour is automatic and learning and reassessment of actions is not necessary.

Imaging studies have shown cortical thinning in the frontostriatal circuits in PD patients with ICDs which may lead to a reduction of “top-down control” and are likely responsible for poor inhibitory control in patients [33] as well as healthy volunteers [34]. Abnormalities in dopamine receptors with dysfunctional activation of autoreceptors in the midbrain and dysfunction of the cortical homeostasis have been shown in PD patients with ICDs [35] and may also contribute to poor decision making and impulsivity [36–38]. Only a few studies have assessed the relationship of ICDs in PD and genetic predisposing factors. A single study found a higher rate of polymorphism in the dopamine receptor 3 (DRD3) gene in PD patients with ICDs compared to those without [39], while other studies did not report any differences in the various candidate gene studies including DRD1, DRD 4, and the dopamine catechol-O-methyl transferase (COMT) [40–43]. Finally, it has been proposed that dyskinesias and ICDs are closely related [44] with similar pattern of oscillatory activity in the subthalamic nucleus [45] and support the notion of continuous dopaminergic stimulation as treatment strategies in these patients.

## 4. ICDs and Sleep

Sleep disturbances, such as sleep fragmentation and daytime sleepiness, have been often described by PD patients with ICDs [8–10]. For example, one study has shown that PD patients with ICDs had poorer sleep than PD controls and healthy volunteers assessed by self-reported PD sleep scales. Furthermore, in this study higher anxiety and depression scores correlated with poorer sleep [9]. Greater nocturnal awakenings, increased frequency of restless legs syndromes, and greater daytime sleepiness are also more common in PD patients with ICDs compared to PD controls [8]. It is, however, unclear whether sleep disturbances increase vulnerability to develop an ICD or are the result of addictive behaviours [8]. For example, it has been described that gamblers can play for days without proper sleep [46]. Furthermore, patients with behavioural addiction may lose track of time and the easy access to internet 24 hours per day is likely contributing to sleep deprivation [46]. Thus, a combination of psychosocial and biological factors plays a key role in sleep disturbances in PD patients with addictive behaviours.

However, the relationship between sleep disturbances and addiction is bidirectional [46–48]. Thus, addiction may also influence so-called clock genes, which are important for sleep-wake cycle regulation. The master clock is located in the suprachiasmatic nucleus of the anterior hypothalamus and clock genes coordinate other brain areas and peripheral organs [48]. Psychoactive drugs as well as behavioural addictions can cause alteration of the expression of these clock genes which in turn lead to sleep disturbances and increase the vulnerability for addictions [48–50]. For example, chronic alcohol intake can lead to a dysfunction of the sleep-wake cycle via disruption of the circadian gene expression [48]. Furthermore, it has been proposed that these clock genes may also be involved in mesolimbic dopaminergic regulation causing neuroplastic changes that may contribute to the development of addiction [48]. In addition, it has been shown that insufficient and mistimed sleep has a major impact on the human transcriptome and affects several distinct molecular pathways [51].

The pathophysiology of sleep deprivation and impulsivity is, however, still poorly understood. It is likely that sleep deprivation leads to a dysfunction of the prefrontal cortex and its connection to the limbic system [52], contributing to a further reduction in top-down inhibitory control in susceptible individuals. In line with this, sleep deprivation in healthy controls can cause impulsive choice possibly due to loss of inhibitory control [53, 54] and upregulation of brain reward networks [55]. Sleep disorders are also prevalent among psychiatric patients or patients with substance abuse as well as prisoners [56, 57] and correlate with increased self-rated aggression and impulsivity [57].

REM sleep behavioural disturbances (RBD) are common in PD and are characterized by loss of the normal REM sleep atonia, enabling patients to “act out” their dreams. Although the pathophysiology is still not completely understood, dysfunction of tonic areas in the lower brainstem is thought to be the key factors of RBD [58]. RBD occurs in approximately 35% to 50% of PD patients [59, 60] and has been associated with male gender, older age, longer disease duration, hyposmia, more advanced PD with motor handicaps, higher levodopa doses, and more psychiatric comorbidity [60, 61]. RBD symptoms either precede, cooccur, or follow parkinsonism. Several studies have shown that those patients who have RBD suffer from more motor and nonmotor symptoms, such as cognitive impairment and autonomic dysfunction, than those without RBD. Some [62, 63] but not all [64] studies have linked RBD with ICD. One study showed that RBD was associated with a more than twofold risk to develop ICD symptoms and a more than fourfold risk to develop pathological gambling [62]. In contrast to the study by Bayard et al. [64], Fantini et al. showed that RBD was associated with a more than twofold risk to develop ICD symptoms [62]. However, in the study by Fantini and colleagues the diagnosis of RBD was based entirely on questionnaires [62] while in the study by Bayard et al. polysomnography to confirm the diagnosis was performed in 30% of patients [64]. RLS is frequent in PD [65–67] although the differential diagnosis is challenging and other causes of restlessness must be excluded [68]. In RLS patients without PD, augmentation and ICDs are increasingly recognized as serious side effects of dopaminergic therapy [69–71].

## 5. Treatment of Sleep Disturbance in PD Patients with ICDs

It is of paramount importance to treat the underlying ICD by reducing the causative agent. In some cases hospital admission is required, due to side effects such as anxiety, irritability, or worsening of motor symptoms [26]. A particular challenge in treating PD patients with ICDs is the dopamine agonist withdrawal syndrome. These withdrawal syndromes resemble those of other drug withdrawal syndromes. Insomnia, panic attacks, dysphoria, fatigue, and depression are commonly seen. Patients often insist to receive a higher amount of medication in order to treat these withdrawal symptoms and are at risk of developing dopamine dysregulation syndrome [26, 72].

As with any sleep disturbances clinicians should first assess whether a primary sleep disorder, such as insomnia, restless legs syndrome, REM sleep behaviour disturbance and periodic limb movements of sleep, sleep apnea, circadian rhythm disturbance, or other factors such as pain, depression, nocturia, dystonia, and difficulty turning in bed are the culprit of poor sleep [73]. In some cases further diagnostic steps, for example, a polysomnography or actigraphy to assess rest-activity cycles over a two-week period, are needed. If a specific sleep disorder is present, it should be treated accordingly. In some patients behavioural recommendations such as the recommendation to avoid long daytime napping can be helpful [74]. There are, however, no guidelines on how to treat sleep disturbances in PD patients with ICDs. Often poor patients' insight and low compliance are major therapeutic challenges.

### 5.1. Nonpharmacological Strategies

Access to credit cards, money, and particularly internet should be restricted [29] not only to improve the addiction but also night time sleep.

Behavioural interventions have been recommended by the American-Academy of Sleep Medicine for all patients who suffer from chronic insomnia [75]. One small randomised controlled study in PD patients without ICDs showed that cognitive behavioural therapy in combination with bright light improved insomnia [76], but these findings need to replicate in larger trials. Patients and their families are advised to consolidate day-night rhythms by timed light exposure and melatonin [77].

### 5.2. Specific Treatment Options

Often nonpharmacological therapies are insufficient in PD patients with ICDs to improve insomnia. The tricyclic antidepressant amitriptyline may be a useful off-label option in some patients, but side effects such as cognitive impairment, dry mouth, and regular heart monitoring are limiting factors. Trazodone, another sedating antidepressant, is sometimes used [78]. Similarly, the presynaptic alpha 2 adrenoreceptor antagonist mirtazapine can be useful to improve insomnia in PD [79].

One randomised controlled trial showed that in PD patients without ICDs melatonin significantly improved subjective sleep quality which, however, could not be demonstrated in polysomnography [80].

Quetiapine has been reported to improve punding, pathological gambling, and compulsive sexual disorder [81], and one preliminary study also showed improvement in insomnia severity [82]. Clozapine can also improve ICDs [83, 84] as well as sleep [85] but carries the potential risk of agranulocytosis.

Benzodiazepines, such as clonazepam, can be used with caution in ICD patients with RBD or in those who continue to suffer from insomnia, but confusion, risk of falling, and sleep apnea limit its use [78].

## 6. Conclusions

Sleep disturbances are very common in PD patients with ICDs but there are no specific guidelines on how to treat these problems. Reduction or complete cessation of dopamine agonists is often necessary to improve the underlying addictive behaviour. Behavioural intervention in combination with pharmacotherapy should be considered in all patients in order to improve night time sleep and ultimately quality of life of the patients and also their partners. Future studies assessing the causal relationship of sleep disturbances and impulsivity in PD as well as randomised controlled studies focussing on treatment strategies for sleep disturbances in PD patients with ICDs are an unmet need.

## Practice Points

- Sleep fragmentation and changes in sleep-wake cycle are common in patients with Parkinson's disease and impulse control disorders.
- Initial steps should focus on treating the underlying behavioural addiction and often include pharmacological and nonpharmacological strategies such as reduction of dopamine agonists, restricted access to computers, or credit cards.
- Therapies with sedative antidepressants, neuroleptic drugs such as quetiapine or clozapine, or benzodiazepines should be considered.
